# Prospective study of hair recovery after (neo)adjuvant chemotherapy with scalp cooling in Japanese breast cancer patients

**DOI:** 10.1007/s00520-021-06168-y

**Published:** 2021-04-02

**Authors:** Shozo Ohsumi, Sachiko Kiyoto, Mina Takahashi, Seiki Takashima, Kenjiro Aogi, Sachie Shimizu, Miyuki Doi

**Affiliations:** Department of Breast Oncology, National Hospital Organization Shikoku Cancer Center, 160 Kou, Minami-umemoto-machi, Matsuyama, Ehime 791-0280 Japan

**Keywords:** Scalp cooling, Alopecia, Hair recovery, Breast cancer, Asian women, Chemotherapy

## Abstract

**Purpose:**

Scalp cooling during chemotherapy infusion to mitigate alopecia for breast cancer patients is becoming widespread; however, studies regarding hair recovery after chemotherapy with scalp cooling are limited. We conducted a prospective study of hair recovery after chemotherapy with scalp cooling.

**Patients and methods:**

One hundred and seventeen Japanese female breast cancer patients who completed planned (neo)adjuvant chemotherapy using the Paxman Scalp Cooling System for alopecia prevention were evaluated for alopecia prevention in our prospective study. We evaluated their hair recovery 1, 4, 7, 10, and 13 months after chemotherapy. Primary outcomes were grades of alopecia judged by two investigators (objective grades) and patients’ answers to the questionnaire regarding the use of a wig or hat (subjective grades).

**Results:**

Of 117 patients, 75 completed scalp cooling during the planned chemotherapy cycles (Group A), but 42 discontinued it mostly after the first cycle (Group B). Objective and subjective grades were significantly better in Group A than in Group B throughout 1 year, and at 4 and 7 months after chemotherapy. When we restricted patients to those with objective Grade 3 (hair loss of > 50%) at 1 month, Group A exhibited slightly faster hair recovery based on the objective grades than Group B. There was less persistent alopecia in Group A than in Group B.

**Conclusions:**

Scalp cooling during chemotherapy infusion for Japanese breast cancer patients increased the rate of hair recovery and had preventive effects against persistent alopecia.

**Supplementary Information:**

The online version contains supplementary material available at 10.1007/s00520-021-06168-y.

## Introduction

Breast cancer is the most common cancer in women worldwide [1]. (Neo)adjuvant chemotherapy is an important part of breast cancer treatment to improve the prognosis. However, the chemotherapeutic agents used in (neo)adjuvant chemotherapy for breast cancer almost always cause severe chemotherapy-induced alopecia, which distresses the patients [2]. Accordingly, scalp cooling during chemotherapy infusion to mitigate alopecia for breast cancer patients has become widespread in European and North American countries. We previously reported the results of alopecia mitigation by scalp cooling during (neo)adjuvant chemotherapy with anthracyclines and/or taxanes for Japanese breast cancer patients in a prospective study, and demonstrated that scalp cooling reduced severe alopecia due to chemotherapy by half [3]. On the other hand, prospective studies regarding hair recovery after chemotherapy with scalp cooling are limited. We conducted a prospective follow-up study of the 117 patients who did not have previous chemotherapy and report their hair recovery.

## Patients and methods

One hundred and twenty-two Japanese female breast cancer patients who completed planned (neo)adjuvant chemotherapy using the Paxman Scalp Cooling System (PAXMAN) for alopecia prevention were evaluated for alopecia in our prospective study. The results of alopecia mitigation were previously reported [3]. In brief, scalp cooling was performed 30 min prior to, during, and 90 min after each chemotherapy infusion. Our previous article included the results 1 month after the last infusion of 4 to 8 cycles of (neo)adjuvant chemotherapy. We reported that 60.7% of the evaluable patients had had hair loss of > 50%, and 86.1% of them almost always used a wig or hat at 1 month after chemotherapy [3]. We then prospectively evaluated hair recovery 4, 7, 10, and 13 months after the completion of chemotherapy. We photographed the heads of the patients from 5 directions, the front, back, both sides, and top, and asked the patients about the use of a wig or hat to conceal alopecia by selecting one of the following answers: not at all, sometimes, and almost always, using a questionnaire at the times mentioned above. Primary outcomes were grades of alopecia judged by two investigators, SO and MD, who were blinded to whether the patients completed scalp cooling throughout the planned chemotherapy according to the WHO grading scale with modification (defined as Grade 0: 0% of hair loss, Grade 1: 1~25%, Grade 2: 26~50%, and Grade 3: > 50%), and patients’ answers to a brief questionnaire asking about the use of a wig or hat (defined as Grade 0: not at all, Grade 1: sometimes, and Grade 2: almost always). We referred to the former as “objective grades” and the latter as “subjective grades.” The objective grades of 1 to 3 at 13 months were defined as persistent alopecia. As reported previously [3], among the 122 patients, 79 completed scalp cooling during the planned chemotherapy cycles, but 43 discontinued it mostly after the first cycle due to several reasons. We excluded 5 patients who received previous chemotherapy before participating in this treatment. We compared the objective and subjective grades of alopecia between the patients who completed scalp cooling (75 patients: Group A) and those who discontinued it (42 patients: Group B).

The Mann-Whitney *U* test and chi-square test were used to examine the significance and *P* values of < 0.05 were considered significant.

This study was approved by the ethics committee of the National Hospital Organization Shikoku Cancer Center in July 2015. This study was performed between September 2015 and September 2019.

## Results

Written informed consent was received from all participants. The mean and median ages at the start of chemotherapy were 50.0 and 49 years, respectively (age range: 28–71). The characteristics of the patients and diseases, and chemotherapeutic and hormonal regimens are shown in Table 1.

**Table 1:** Characteristics of the patients and diseases, and chemotherapeutic and hormonal regimens (*n* = 117)

Age at the start of chemotherapy (years)	Mean 50.0 (range 28–71)	Median 49 years
Clinical or pathological stage	Stage I	43
	Stage IIA	34
	Stage IIB	16
	Stage IIIA	12
	Stage IIIB	3
	Stage IIIC	5
	Unknown	4
Estrogen receptor status	Positive	79
	Negative	38
Progesterone receptor status	Positive	67
	Negative	50
HER2 status	Positive	23
	Negative	94
Timing of chemotherapy	Preoperative	14
	Postoperative	103
Chemotherapy regimen	TC (DTX 75 mg/m^2^ + CPA 600 mg/m^2^) × 4	82
	AC (A 60 mg/m^2^ + CPA 600 mg/m^2^) or EC (E 90 mg/m^2^ + CPA 600 mg/m^2^) × 4 followed by taxanes (DTX 75 mg/m^2^ or Paclitaxel 175 mg/m^2^) × 4	33
	EC (E 90 mg/m^2^ + CPA 600 mg/m^2^) × 4	1
	(DTX 75 mg/m^2^ + CBDCA AUC 6) × 4 followed by T-DM1 × 4	1
Adjuvant endocrine therapy at 13 months	Tamoxifen	51
	Aromatase inhibitor	22
	LH-RH agonist + aromatase inhibitor	6
	None	38

*DTX* docetaxel, *CPA* cyclophosphamide, *AC* doxorubicin and cyclophosphamide, *EC* epirubicin and cyclophosphamide, *CBDCA* carboplatin

**Fig. 1:**
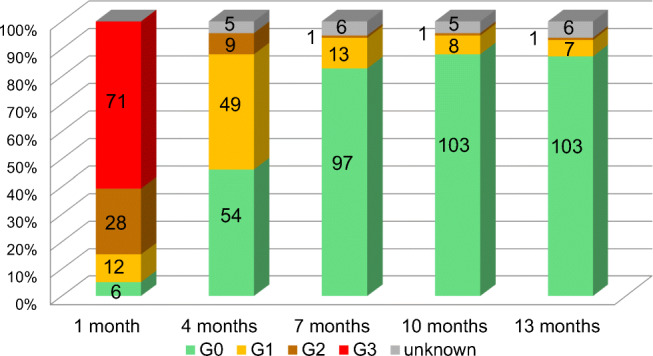
Objective grades of alopecia in all patients (*n* = 117)

In all 117 patients, objective Grades at 1 month after the completion of chemotherapy were Grade 0 in 6 patients (5.1%), Grade 1 in 12, Grade 2 in 28, and Grade 3 in 71. The changes over time in objective Grade 0 were as follows: at 4 months: Grade 0 in 54 (48.2%); at 7 months: Grade 0 in 97 (87.4%); at 10 months: Grade 0 in 103 (92.0%); at 13 months: Grade 0 in 103 (92.8%) (Fig. 1). Alopecia at 13 months, which was defined as persistent alopecia, was observed in 8 patients (7.2%) (median age 57.5: age range 28–68). Of 8 patients, 3 received TC (docetaxel + cyclophosphamide) × 4, 4 received AC (doxorubicin + cyclophosphamide) (or EC) (epirubicin + cyclophosphamide) × 4 followed by taxanes × 4, and 1 received EC × 4 (Supplementary Table 1).

**Fig. 2:**
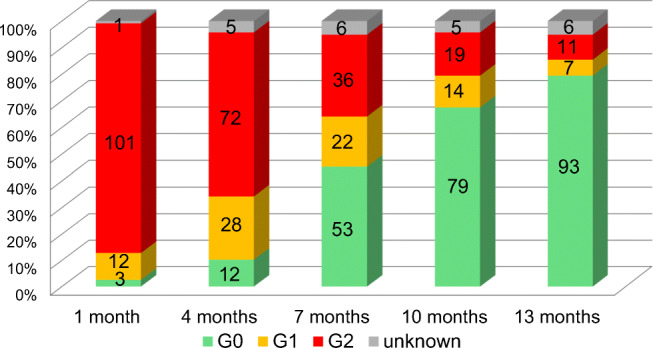
Subjective grades of alopecia in all patients (*n* = 117)

On the other hand, subjective Grades at 1 month were Grade 0 in 3 patients (2.6%), Grade 1 in 12, Grade 2 in 101, and unknown in 1. The changes over time in subjective Grade 0 were as follows: at 4 months: Grade 0 in 12 (10.7%); at 7 months: Grade 0 in 53 (47.7%); at 10 months: Grade 0 in 79 (70.5%); and at 13 months: Grade 0 in 93 (83.8%) (Fig. 2).

**Fig. 3:**
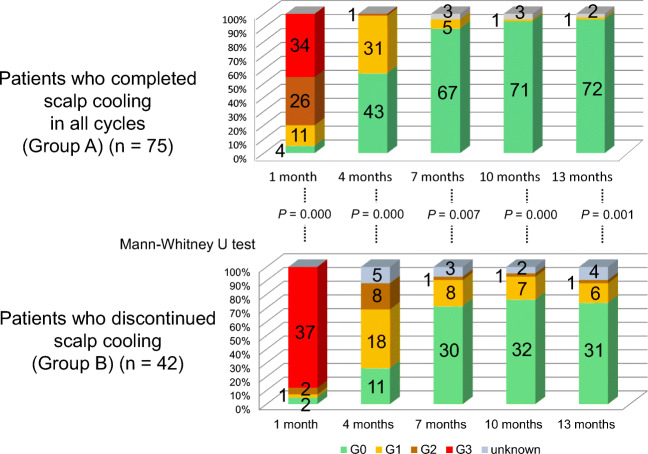
Objective grades of alopecia. Comparison between Groups A (*n* = 75) and B (*n* = 42)

**Fig. 4:**
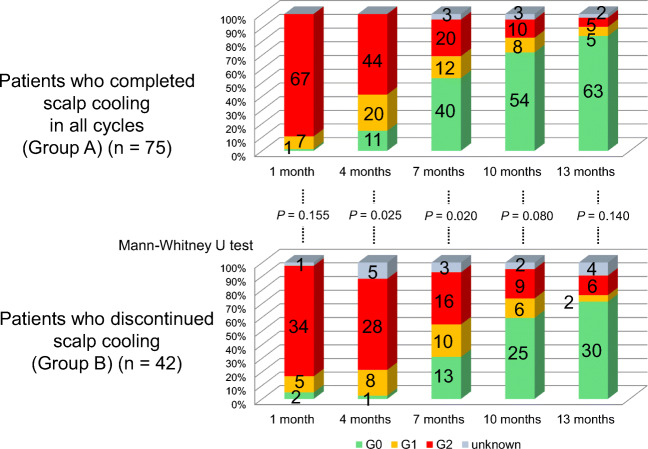
Subjective grades of alopecia. Comparison between Groups A (*n* = 75) and B (*n* = 42)

When we compared Group A with Group B, there were no differences in the age, chemotherapeutic regimens, or adjuvant endocrine therapy (Table 2). As for hair recovery, the objective grades were significantly lower in Group A than in Group B throughout 1 year after chemotherapy (*P* = 0.000 at 1 month, *P* = 0.000 at 4 months, *P* = 0.007 at 7 months, *P* = 0.000 at 10 months, and *P* = 0.001 at 13 months) (Fig. 3), and the subjective grades were significantly better in Group A than in Group B at 4 and 7 months (*P* = 0.025, and 0.020, respectively) (Fig. 4) . Regarding persistent alopecia, there were 1 (1.4%) and 7 patients (18.4%) who had persistent alopecia in Groups A and B, respectively (*P* = 0.004 by the chi-square test). The patient with persistent alopecia in Group A received TC × 4, but was not administered hormonal agents, whereas of the patients with persistent alopecia in Group B, 3 received an aromatase inhibitor, 2 received tamoxifen at 13 months, and 2 were not administered hormonal agents (Supplementary Table 1).

**Table 2:** Comparison of the age, chemotherapeutic regimens, and adjuvant endocrine therapy between Groups A and B

		Patients who completed the scalp cooling in all cycles (Group A) (*n* = 75)	Patients who discontinued the scalp cooling (Group B) (*n* = 42)	*P* value
Age at the start of chemotherapy (years)	Mean (age range)	50.3 (28–70)	49.5 (28–71)	0.67
	Median	49	49	
Chemotherapy regimen	TC (DTX + CPA) × 4	55 (73.3%)	27 (64.3%)	0.34
	AC or EC × 4 followed by taxanes × 4	20 (26.6%)	13 (31.0%)	
	EC × 4	0	1 (2.4%)	
	(DTX + CBDCA) × 4 followed by T-DM1 × 4	0	1 (2.4%)	
Adjuvant endocrine therapy at 13 months	Tamoxifen	33 (44.0%)	18 (42.9%)	0.96
	Aromatase inhibitor	13 (17.3%)	9 (21.4%)	
	LH-RH agonist + aromatase inhibitor	4 (5.3%)	2 (4.8%)	
	None	25 (33.3%)	13 (31.0%)	

*DTX* docetaxel, *CPA* cyclophosphamide, *AC* doxorubicin and cyclophosphamide, *EC* epirubicin and cyclophosphamide, *CBDCA* carboplatin. The *t* test and chi-square test were used

When we restricted patients to those with objective Grade 3 at 1 month (*n* = 71), both objective and subjective grades gradually improved, as shown in Supplementary Figs. 1 and 2. When we compared objective Grade 3 patients in Group A (*n* = 34) and Group B (*n* = 37), Group A exhibited slightly faster hair recovery based on the objective grades than Group B, and the grades were significantly better at 10 and 13 months (*P* = 0.016 and 0.045, respectively) (Supplementary Fig. 3). On the other hand, there were no differences between Groups A and B in subjective grades throughout 1 year after chemotherapy (Supplementary Fig. 4).

As hormonal agents may have affected the results, we compared the patients who were receiving endocrine therapy with those who were not. At 13 months after chemotherapy, 5 Group A and B patients who were receiving endocrine therapy had objective Grade 1 (5/74, 6.8%), and 2 and 1 who were not had objective Grade 1 and objective Grade 2, respectively (3/37, 8.1%) (*P* = 0.897).

## Discussion

Chemotherapy is one of the major treatments for breast cancer. It is used to improve the disease-free and overall survival in the (neo)adjuvant setting because an improved prognosis was demonstrated in many prospective randomized trials and meta-analyses [4–6]. However, it causes severe alopecia, especially regimens with anthracyclines and/or taxanes as key drugs. Patients may refuse (neo)adjuvant chemotherapy because of alopecia [2, 7, 8]. If chemotherapy-induced alopecia is prevented or reduced, patients with breast cancer will be able to receive (neo)adjuvant chemotherapy more easily.

Many methods have been applied to prevent alopecia during chemotherapy and a meta-analysis of studies regarding chemotherapy-induced alopecia revealed that scalp cooling during chemotherapy infusion is the most effective [9]. However, it cannot completely prevent alopecia. Studies examining the effectiveness of alopecia with scalp cooling, including ours, revealed that it reduces the number of patients who experience hair loss of > 50% by nearly half [3, 10–12].

We hypothesized that scalp cooling protects hair follicle cells from long-term damage, thereby increasing the rate of hair recovery and preventing persistent alopecia. Therefore, we conducted this prospective study of hair recovery after chemotherapy with scalp cooling in patients who participated in our prospective study to examine alopecia mitigation. In our previous single-arm prospective study, 122 patients completed the planned chemotherapy and were evaluable for alopecia. Although they used the cooling cap for free during the first cycle of chemotherapy, they were required to purchase it (approximately US$ 1,130) for the following cycles in the study. Seventy-nine of 122 patients completed the scalp cooling during chemotherapy, but the remaining 43 patients decided to discontinue it mostly after the first cycle. Some patients may have discontinued it because of less preventive effects against alopecia than expected, but at least 28 (65.1%) decided to discontinue within 10 days after the first infusion of chemotherapy, i.e., before the start of alopecia. Although our previous study had a single arm, the 43 patients who discontinued scalp cooling were regarded as a control group, Group B.

To our knowledge, there are two similar prospective studies that examined hair recovery after chemotherapy with scalp cooling. One is the COOLHAIR study, which was a prospective randomized study comparing scalp cooling with the DigniCap System (CAP arm) and no scalp cooling (NoCAP arm) [13]. They evaluated hair recovery at 3 and 6 months after chemotherapy in both arms. They reported rates of hair regrowth > 75% judged by the patients themselves (CAP arm: 66.7%, *n* = 12 vs. NoCAP arm: 75.0%, *n* = 12 at 3 months, and CAP arm: 94.7% vs. NoCAP arm: 100% at 6 months). The other study was the HOPE study, which was a prospective non-randomized study [14]. They evaluated hair recovery only up to 12 weeks after completing chemotherapy. They used PAXMAN for scalp cooling, and reported faster hair recovery in the PAXMAN group (*n* = 28) than in the control group (*n* = 12). They compared the patients with hair loss of greater than 50% or those requiring a wig, namely, Grade 2, at 3 weeks after the final cycle of chemotherapy between the PAXMAN group (*n* = 20) and control group (*n* = 12). The rates of hair recovery to Grade 0 (no hair loss) at 12 weeks were 25% (5/20) and 8.3% (1/12) in the PAXMAN group and control group, respectively. The proportion of patients recovering from Grade 2 alopecia was significantly higher in the PAXMAN group than in the control group (*P* < 0.05).

Our prospective follow-up study was the first to closely follow the patients for a long time, up to 13 months, after chemotherapy. Objective hair recovery was faster in Group A than in Group B in all patients and in those with objective Grade 3 alopecia at 1 month. Scalp cooling may have reduced the damage to the hair follicle cells even if the patients developed objective Grade 3 alopecia during the chemotherapy period. On the other hand, there were no large differences in subjective grades over 1 year after chemotherapy between Groups A and B. The most probable reason for the fewer differences in the subjective grades than in the objective grades is as follows: as Japanese women are generally sensitive about their appearance, even if there was a slight reduction in hair volume, they likely continued to use a wig or hat until the hair recovered to its original volume.

Historically, chemotherapy-induced alopecia was regarded as an adverse event that recovers completely after breast cancer treatment. However, several reports described persistent alopecia after chemotherapy for breast cancer, especially after using docetaxel [15, 16]. Kluger et al. followed 20 breast cancer patients who mainly received sequential fluorouracil/epirubicin/cyclophosphamide (FEC) and docetaxel, and developed permanent alopecia, which was defined as absent or incomplete hair regrowth at 6 months or longer post-chemotherapy, for 15.6 months prospectively [17]. They reported no significant regrowth on the scalp and the patients had a significant decrease in their quality of life. Kang et al. prospectively evaluated hair recovery in 61 breast cancer patients who received adjuvant chemotherapy up to 3 years after chemotherapy, and reported that the proportions of patients who had absent or incomplete hair growth at 6 months and 3 years were 39.5% and 42.3%, respectively [18]. Martin et al. assessed Grade 2 persistent alopecia, defined as complete alopecia that requires a wig at least after 18 months from the end of adjuvant chemotherapy [19]. They found 36 patients with Grade 2 persistent alopecia among 358 who received a cumulative dose of docetaxel of 400 mg/m^2^ or more. They also reported that the Grade 2 persistent alopecia disappeared after using a cold cap, ELASTO-GEL, during chemotherapy infusion.

Persistent alopecia, defined as hair loss at 13 months in this study, was more frequently observed in Group B than in Group A (18.4% vs. 1.4%). This difference was significant (*P* = 0.004 by the chi-square test). Although adjuvant endocrine therapy, especially with aromatase inhibitors, causes hair loss to some extent [20], there was no difference in the use of hormonal agents between the groups (Table 2). Persistent alopecia even in Group B was only hair loss of 50% or less. This may have been caused by the cumulative dose of docetaxel. Martin et al. did not observe complete alopecia after at least 18 months in patients who received < 400 mg/m^2^ of docetaxel or other chemotherapy regimens not containing docetaxel [19]. As we used 75 mg/m^2^ of docetaxel in TC and AC or EC followed by docetaxel, the cumulative dose was 300 mg/m^2^. The low rate of persistent alopecia may also have been caused by racial differences and the use of cyclophosphamide.

The preventive effects against persistent alopecia by scalp cooling strongly support the hypothesis that it reduces the irreversible damage to hair follicle cells by chemotherapeutic agents. Persistent alopecia is considered to be one of the most distressing long-term side effects of chemotherapy. Therefore, it is worth continuing even if patients experience alopecia after the first cycle of chemotherapy with scalp cooling.

Our study has several strong points. First, it was performed prospectively. Second, the number of patients was relatively large. Third, few patients dropped out from follow-up.

There are however limitations. First, although we set a control group, Group B, our study was not conducted in a randomized manner. Therefore, when Groups A and B were compared, there was a bias. However, as shown in Table 2, there were no differences in regimens delivered between the two groups, and as we reported previously, the reason why most patients in Group B decided to discontinue scalp cooling was not a lower-than-expected efficacy of alopecia prevention by scalp cooling. Second, we did not have dermatologists in our study group. The dermatological methods of examining the hair may have clarified the differences between the objective and subjective grades. In addition, it was not possible to exclude other alopecia etiologies because of the lack of further evaluation.

## Conclusion

We concluded that scalp cooling during chemotherapy infusion for Japanese breast cancer patients increased the rate of hair recovery and reduced the rate of persistent alopecia to almost zero.

## Supplementary Information

ESM 1 Supplementary Table 1 Patients who had alopecia at 13 months after chemotherapy. Supplementary Fig. 1 Objective grades of alopecia in patients with objective Grade 3 alopecia at 1 month after chemotherapy (*n* = 71). Supplementary Fig. 2 Subjective grades of alopecia in patients with objective Grade 3 alopecia at 1 month after chemotherapy (*n* = 71). Supplementary Fig. 3 Objective grades of alopecia in patients with objective Grade 3 alopecia at 1 month after chemotherapy. Comparison between Groups A (*n* = 34) and B (*n* = 37). Supplementary Fig. 4 Subjective grades of alopecia in patients with objective Grade 3 alopecia at 1 month after chemotherapy. Comparison between Groups A (*n* = 34) and B (*n* = 37) (PPTX 322 kb)

## Data Availability

Our previous paper was published in Supportive Care in Cancer 2021.

## Abbreviations

AC: Doxorubicin and cyclophosphamide
CBDCA: Carboplatin
CPA: Cyclophosphamide
DTX: Docetaxel
EC: Epirubicin and cyclophosphamide
PAXMAN: The Paxman Scalp Cooling System
PTX: Paclitaxel
TC: Docetaxel and cyclophosphamide

## Code availability

StatMateV software package.
