# Supplementary material for: Prospective study of hair recovery after (neo)adjuvant chemotherapy with scalp cooling in Japanese breast cancer patients
Source: Support Care Cancer. 2021 Apr 2;29(10):6119–25. doi: 10.1007/s00520-021-06168-y (PMC8410694; doi:10.1007/s00520-021-06168-y)
Supplement: Supplementary file 1 — Supplementary Table 1 Patients who had alopecia at 13 months after chemotherapy. Supplementary Fig. 1 Objective grades of alopecia in patients with objective Grade 3 alopecia at 1 month after chemotherapy (n = 71). Supplementary Fig. 2 Subjective grades of alopecia in patients with objective Grade 3 alopecia at 1 month after chemotherapy (n = 71). Supplementary Fig. 3 Objective grades of alopecia in patients with objective Grade 3 alopecia at 1 month after chemotherapy. Comparison between Groups A (n = 34) and B (n = 37). Supplementary Fig. 4 Subjective grades of alopecia in patients with objective Grade 3 alopecia at 1 month after chemotherapy. Comparison between Groups A (n = 34) and B (n = 37) (PPTX 322 kb) [file 520_2021_6168_MOESM1_ESM.pptx]

## Slide 1
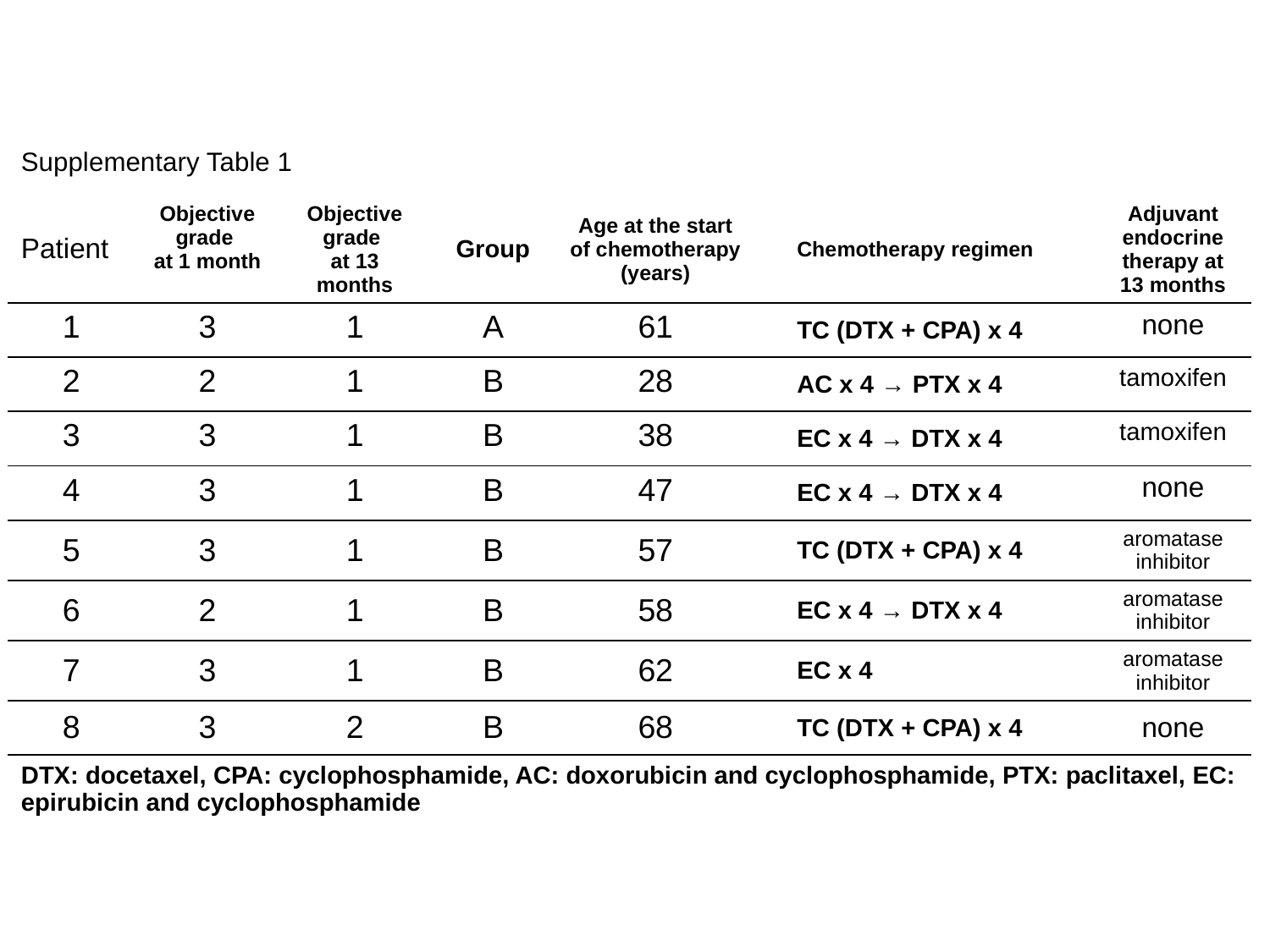

| Supplementary Table 1 | | | | | | | |
| --- | --- | --- | --- | --- | --- | --- | --- |
| Patient | Objective grade at 1 month | Objective grade at 13 months | Group | Age at the start of chemotherapy (years) | | Chemotherapy regimen | Adjuvant endocrine therapy at 13 months |
| 1 | 3 | 1 | A | 61 | | TC (DTX + CPA) x 4 | none |
| 2 | 2 | 1 | B | 28 | | AC x 4 → PTX x 4 | tamoxifen |
| 3 | 3 | 1 | B | 38 | | EC x 4 → DTX x 4 | tamoxifen |
| 4 | 3 | 1 | B | 47 | | EC x 4 → DTX x 4 | none |
| 5 | 3 | 1 | B | 57 | | TC (DTX + CPA) x 4 | aromatase inhibitor |
| 6 | 2 | 1 | B | 58 | | EC x 4 → DTX x 4 | aromatase inhibitor |
| 7 | 3 | 1 | B | 62 | | EC x 4 | aromatase inhibitor |
| 8 | 3 | 2 | B | 68 | | TC (DTX + CPA) x 4 | none |
| DTX: docetaxel, CPA: cyclophosphamide, AC: doxorubicin and cyclophosphamide, PTX: paclitaxel, EC: epirubicin and cyclophosphamide | | | | | | | |

## Slide 2
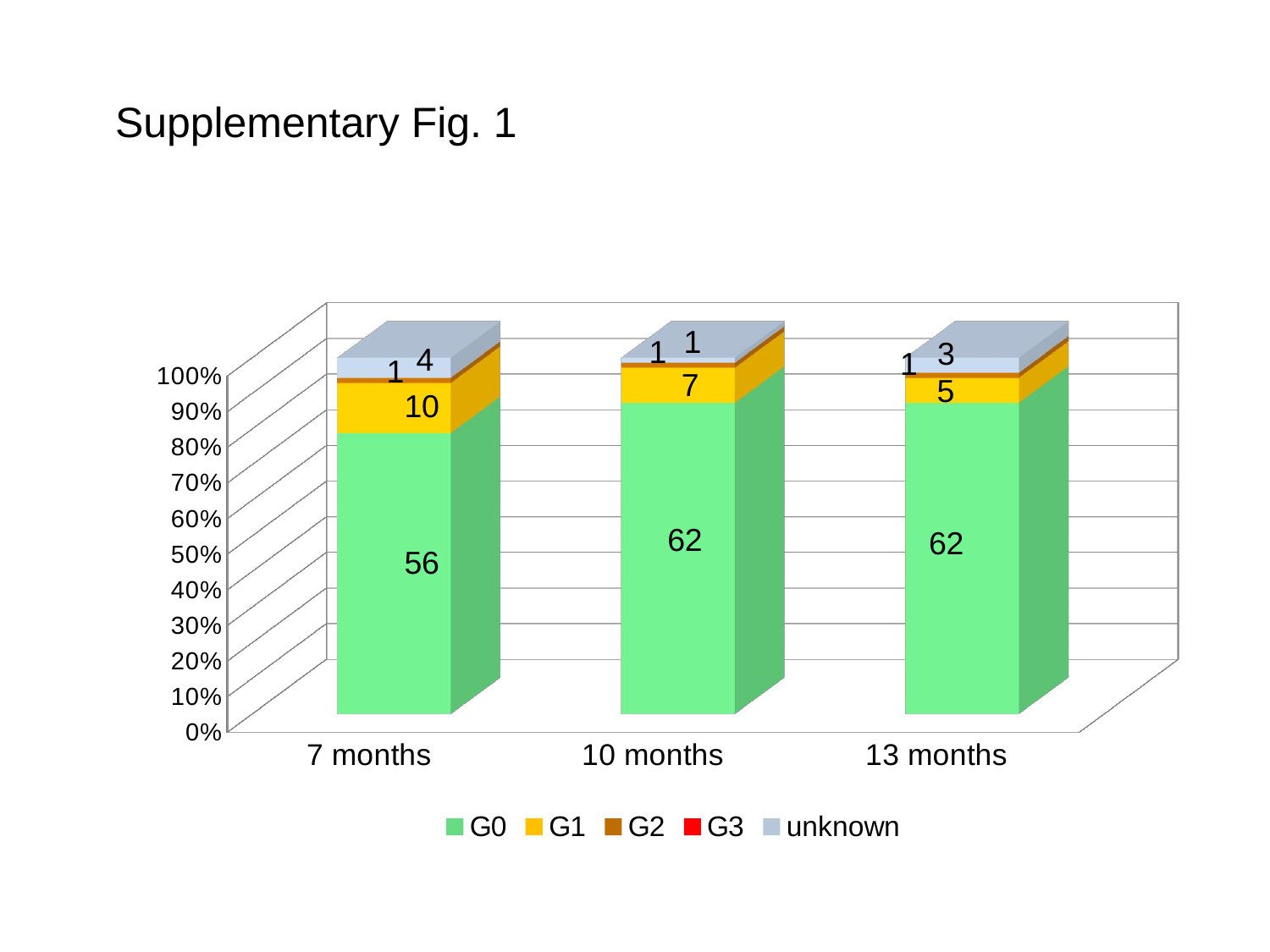

Supplementary Fig. 1
[unsupported chart]
1
1
3
4
1
1
7
5
10
62
62
56

## Slide 3
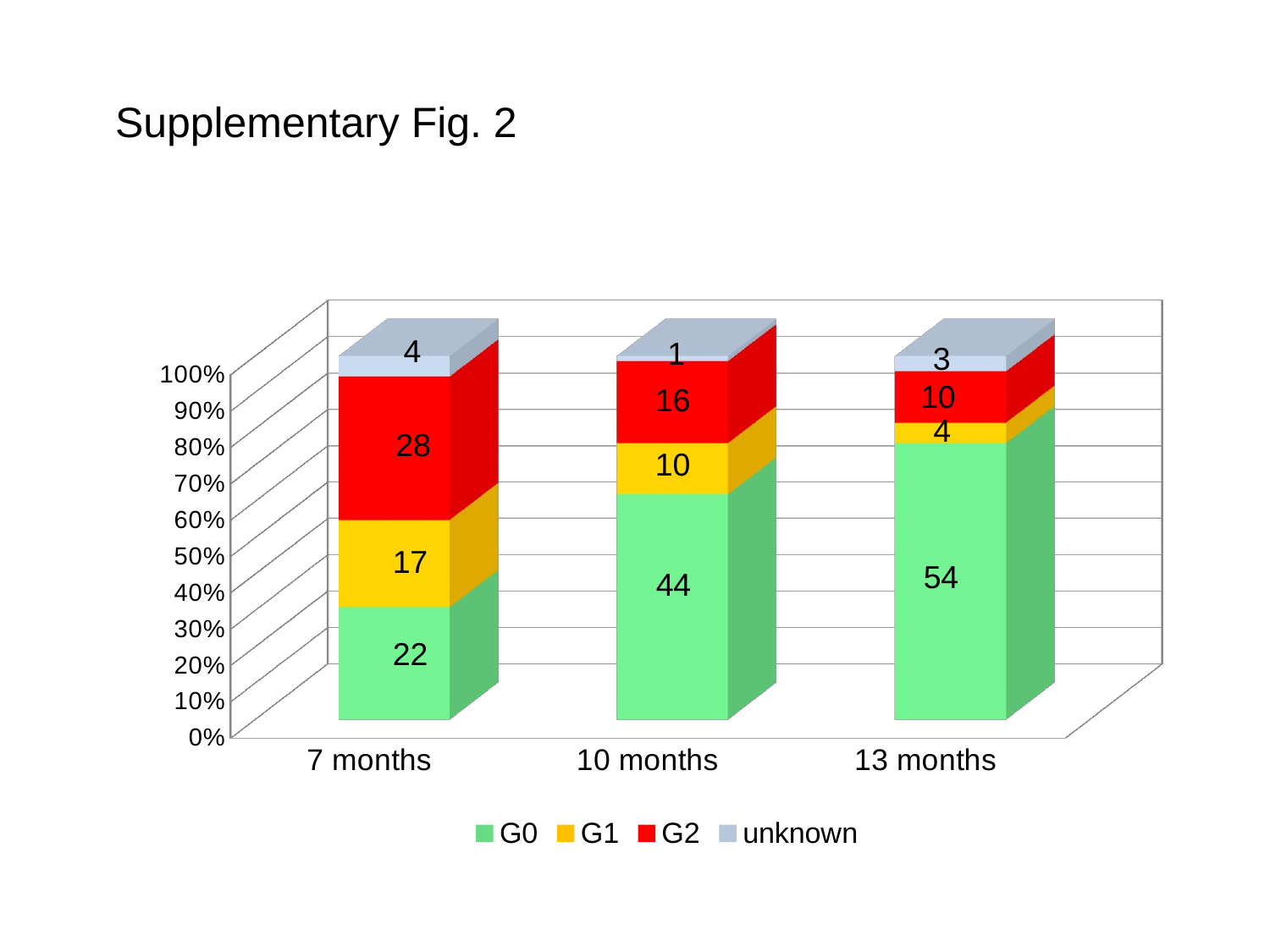

Supplementary Fig. 2
[unsupported chart]
4
1
3
10
16
4
28
10
17
54
44
22

## Slide 4
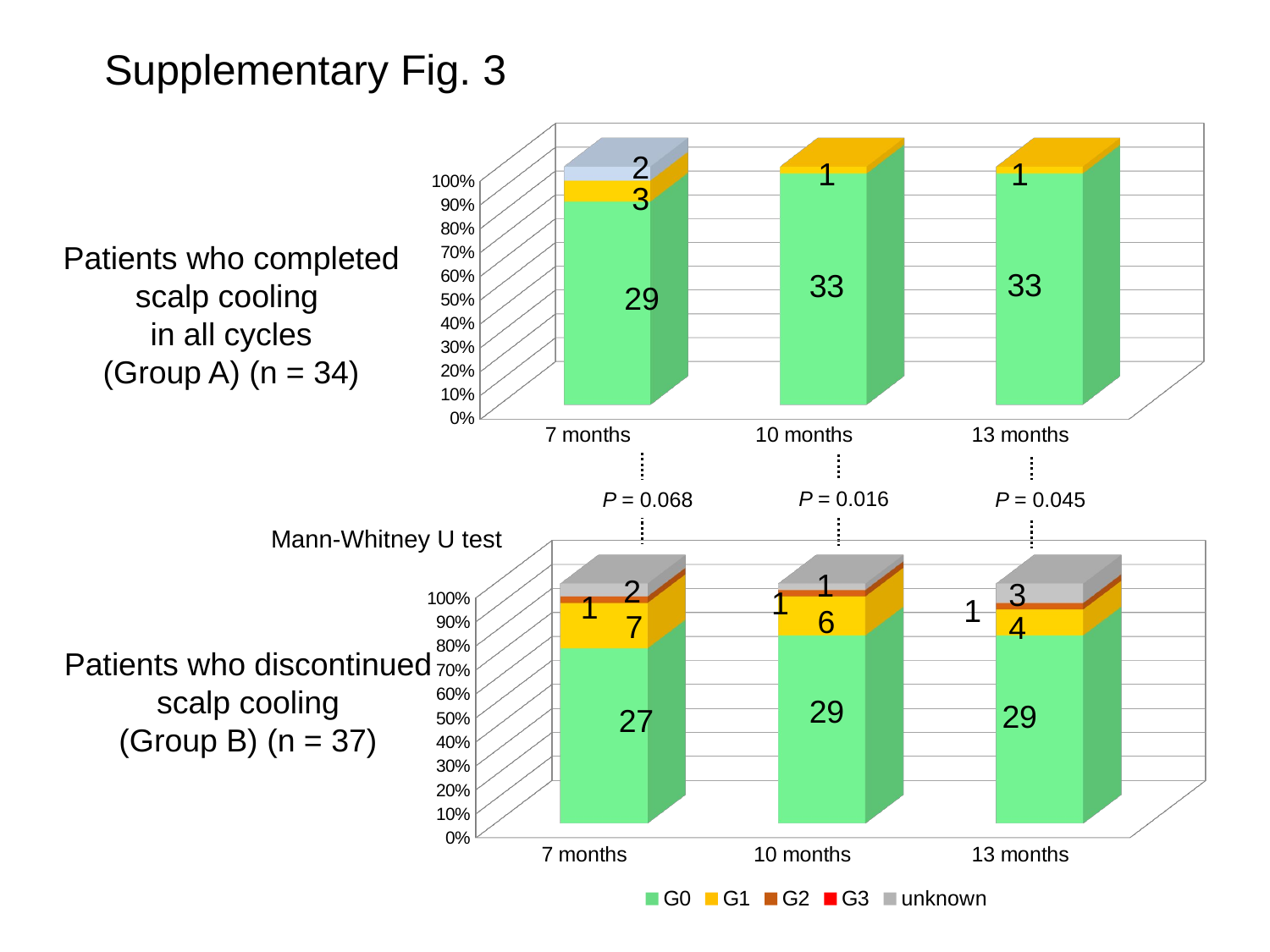

Supplementary Fig. 3
[unsupported chart]
2
1
1
3
Patients who completed
scalp cooling
in all cycles
(Group A) (n = 34)
33
33
29
P = 0.016
P = 0.068
P = 0.045
Mann-Whitney U test
[unsupported chart]
1
2
3
1
1
1
6
7
4
Patients who discontinued
scalp cooling
(Group B) (n = 37)
29
29
27

## Slide 5
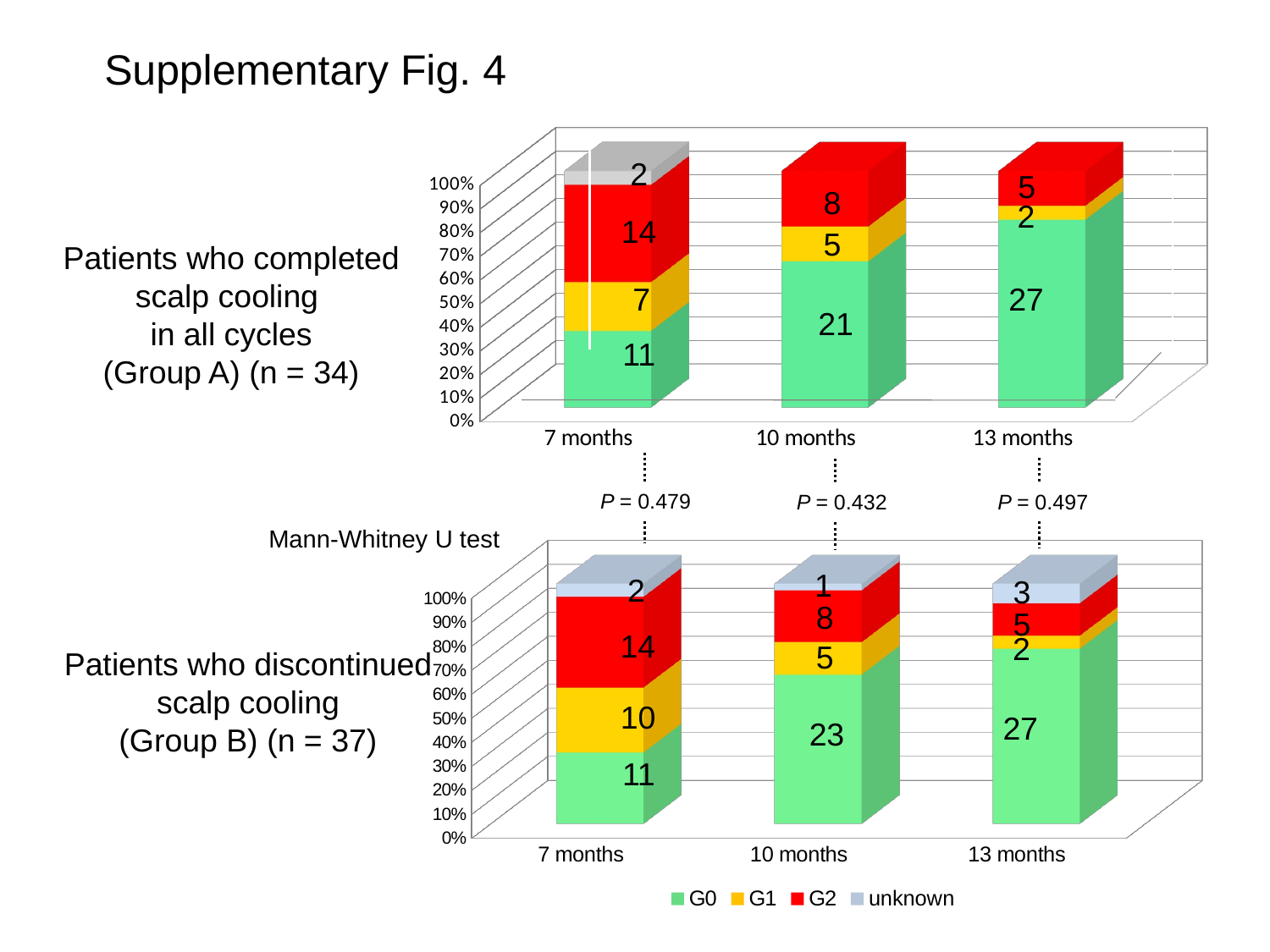

Supplementary Fig. 4
[unsupported chart]
2
5
8
2
14
5
Patients who completed
scalp cooling
in all cycles
(Group A) (n = 34)
27
7
21
11
P = 0.479
P = 0.432
P = 0.497
Mann-Whitney U test
[unsupported chart]
1
2
3
8
5
14
2
5
Patients who discontinued
scalp cooling
(Group B) (n = 37)
10
27
23
11
